# Mitigation of tartrazine induced histopathological and genotoxic effects in *Coturnix coturnix japonica* using green synthesized silver nanoparticles

**DOI:** 10.1016/j.psj.2026.106767

**Published:** 2026-03-13

**Authors:** Samra, Shabana Naz, Maryam Fatima, Fiza Abbas, Ulfat Zahra, Rasha Alonaizan, Hafsa Saeed, Sania Satti, Rifat Ullah Khan, Ala Abudabos, Ali R. Al Sulaiman, Raed Al-Atiyat, Sohail Ahmad, Ibrahim A. Alhidary

**Affiliations:** a Department of Zoology, Government College University, Faisalabad, Pakistan; b Department of Zoology, College of Sciences, King Saud University, Riyadh, Saudi Arabia; c Physiology Lab, College of Veterinary Sciences, Faculty of Animal Husbandry and Veterinary Sciences, The University of Agriculture, Peshawar, Pakistan; d Department of Food and Animal Sciences, College of Agriculture, Tennessee State University, Nashville, TN 37209, USA; e Environmental Protection Technologies Institute, Sustainability and Environment Sector, King Abdulaziz City for Science and Technology, P.O. Box 6086, Riyadh 11442, Saudi Arabia; f Molecular Genetics, breeding and Biotechnology, Animal Sci. Dep., Agriculture Faculty, Mutah University, Karak, Jordan; g Islamic University of Afghanistan, Kabul, Afghanistan; h Department of Animal Production, College of Food and Agriculture Sciences, King Saud University, Riadh 11451, Saudi Arabia

**Keywords:** AgNPs, *Azadirachta indica*, Histopathology, DNA Damage

## Abstract

Silver nanoparticles (Ag-NPs) are widely used across various fields due to their antimicrobial properties; however, their potential toxicity remains a concern. In this current study, Ag-NPs were synthesized with neem extract and determined through UV-Vis spectroscopy. Fourier-transform infrared spectroscopy (FTIR), and X-ray diffraction (XRD) to confirm their structural and chemical properties. The study aimed to evaluate the effects of tartrazine on histopathology, and DNA damage in Japanese quails. A total of 336 one-day-old Japanese quails were classified into seven experimental groups as follows: Group 1 served as the control and was fed only a basic diet, while Group 2 received 10 mg/kg low dose of tartrazine, Group 3 received 20 mg/kg body weight of tartrazine, Group 4 administered with tartrazine low (10mg/kg) and AgNPs low (4 mg/kg) dose group, Group 5 received tartrazine low and AgNPs high (8 mg/kg) dose, Group 6 received tartrazine high and AgNPs low dose and Group 7 received tartrazine high and AgNPs high dose via oral gavage on daily basis basis until 45 days of age. Our results revealed that birds administered 20 mg/kg of tartrazine had significantly higher histopathological changes and DNA. These results suggest that group with tartrazine low and AgNPs high dose (group 5) has results near to control which shows that AgNPs effectively mitigated the effects caused by tartrazine as compare to group treated with high tartrazine dose.

## Introduction

Nanotechnology refers to the study and development of materials at the nanoscale (1–100 nm), enabling the fabrication of structures with unique physicochemical properties distinct from bulk materials (Akram et al., 2025; Nile et al., 2020). Nanotechnology encompasses practical applications operating at nanoscale dimensions and has emerged as a multidisciplinary field integrating nanoscience, nanochemistry, nanophysics, nanomaterials, nanoelectronics, and nanobiotechnology (Al-Gheffari et al., 2024; Al-Suwailem et al., 2024; Naz et al., 2024). Nanomaterials can be engineered with specific functional properties, making them valuable tools for innovative technologies in healthcare, agriculture, food systems, and environmental management (Bhushan et al., 2017; Madkour et al., 2019; Rawat et al., 2023; Şimsek et al., 2025). Because of their high surface-area-to-volume ratio and enhanced reactivity, nanomaterials have attracted increasing attention in biomedical and toxicological applications.

Silver nanoparticles (AgNPs) have gained considerable interest due to their strong antimicrobial activity and favorable physical characteristics, leading to widespread use in water purification, medical devices, and disinfectants (Gong et al., 2007; Rasool et al., 2024). Owing to their distinctive physicochemical properties, AgNPs are considered among the most promising nanomaterials in biomedicine (Sadan et al., 2025). They have been investigated as antimicrobial and anticancer agents, vaccine adjuvants, biosensors, and wound-healing promoters (Usman et al., 2025; Xu et al., 2020). However, biological responses to nanoparticles are strongly influenced by synthesis methods and surface chemistry, highlighting the importance of environmentally safe production strategies.

Environmentally friendly synthesis of AgNPs has recently emerged as a rapidly expanding area of nanotechnology. Green synthetic chemistry involves the development of nanoparticle synthesis processes that avoid toxic reagents and minimize environmental contamination (Al-Khalaifah et al., 2025a, 2025b; Nie et al., 2023; Ahsan et al., 2024). Plant-mediated synthesis provides a simple and reliable method for producing biocompatible nanomaterials while adhering to green chemistry principles. Numerous plant extracts have been used for AgNP synthesis (Al-Khalaifah et al., 2025a, 2025b), including *Azadirachta indica* (neem), a medicinal plant known for antibacterial, antiviral, antifungal, and anti-inflammatory properties (Khan et al., 2022). Neem-based synthesis enhances sustainability and may improve nanoparticle biocompatibility, although concerns regarding nanoparticle toxicity remain (Fatima et al., 2025). Because of its extensive therapeutic potential, neem has been described as the “tree of the twenty-first century” (Kumar et al., 2015).

Parallel to nanotechnology advancements, environmental and biological concerns related to synthetic dyes have increased. Industrialization has contributed to environmental contamination through dye-containing effluents, many of which are nonbiodegradable and hazardous to ecosystems (Hegazy et al., 2023). Tartrazine, a widely used azo dye applied in foods, pharmaceuticals, and cosmetics, is highly soluble and persistent, raising safety concerns for humans and animals (Jodat et al., 2014). Although several physicochemical approaches such as adsorption, photocatalytic degradation, electrochemical oxidation, and electro-Fenton reactions have been explored for dye removal (Akbarzadeh et al., 2023), exposure risks remain due to its continued widespread use (Abbas et al., 2026).

Growing experimental evidence indicates that tartrazine may induce biological toxicity across animal models. Studies in mammals have reported hepatic and renal tissue alterations following exposure to synthetic food dyes (Shathi et al., 2023). Importantly, avian-based investigations also demonstrate susceptibility to tartrazine toxicity. El-Borm et al. (2019) reported hepatotoxic and nephrotoxic effects in chick embryos exposed to tartrazine and sunset yellow dyes, while El-Borm et al. (2020) demonstrated DNA damage and disruption of liver and kidney cell cycles in avian embryos. These findings highlight the need for further toxicological evaluation in poultry species.

Nanoparticles have also been investigated for environmental dye removal due to their high adsorption capacity and catalytic properties (Marimuthu et al., 2025). Silver nanoparticles exhibit photocatalytic activity capable of degrading various dyes, and green-synthesized AgNPs have been shown to degrade approximately 55–60% of tartrazine under experimental conditions (Khan et al., 2023). Beyond environmental remediation, evaluating whether such nanoparticles can mitigate biological toxicity caused by dye exposure represents an emerging research direction.

Japanese quails are considered an ideal experimental model due to their small size, rapid reproduction, adaptability, and importance in poultry production systems (Abbass et al., 2026). Their established use in toxicological and nutritional studies makes them suitable for assessing both health risks and potential protective interventions relevant to poultry science and food safety.

Therefore, the objective of the present study was to green-synthesize and characterize silver nanoparticles using *Azadirachta indica* extract and to evaluate their protective potential against tartrazine-induced toxicity in Japanese quails. Specifically, this study investigated histopathological alterations and DNA damage associated with tartrazine exposure and assessed whether neem-derived AgNPs could mitigate these adverse effects in a dose-dependent manner.

## Materials and methods

### Synthesis of green silver nanoparticles (AgNPs)

After washing the leaves with deionized water and letting them air dry at room temperature, the surfaces were scrubbed with running tap water to get rid of dirt and other contaminated organic materials. A 250 mL conical flask filled with 50 mL deionized water held about 15 g of finely chopped leaves, which were then boiled in a water bath for 25 minutes at 60°C. After cooling to ambient temperature, the extract was vacuum-filtered through Whatman filter and kept for later use at 4°C. An Erlenmeyer flask was filled with 100 mL of a 1 mM silver nitrate (AgNO3) solution. 50 mL of 1 mM aqueous AgNO_3_ solution was mixed with 10 mL of *A. indica* (neem) leaf extract at room temperature, and the mixture was constantly stirred for 20 minutes to create the Ag-NPs. The resulting combination was kept at room temperature in a dark environment to stop the silver nitrate from auto-oxidizing. The solution's color changed from reddish to dark brown, signifying the synthesis of silver nanoparticles (Ag-NPs), and UV-visible spectroscopy was used to confirm the creation of Ag-NPs. The UV–Vis spectrum exhibited a characteristic surface plasmon resonance (SPR) absorption peak at approximately 420 nm, confirming the formation of silver nanoparticles. The Ag-NPs solution was centrifuged for 15 minutes at 2000 rpm after 24 hours, and the pellets that were produced were then dried for 24 hours at 100°C in an oven (Kumari et al., 2022).

The characterization of synthesized AgNPs was done by using UV visible spectrophotometer (Hitachi U-2800), which measures absorbance between 300 and 600 nm to track the development of nanoparticles (Ahmed et al., 2016). The maximum absorption wavelength (λmax) observed at ∼420 nm corresponds to the typical SPR band of spherical silver nanoparticles within the 400–450 nm range, further verifying nanoparticle formation. Utilizing PerkinElmer's Fourier Transform Infrared (FTIR) spectroscopy, the functional groups responsible for stabilizing the Ag-NPs were identified. At a resolution of 4 cm⁻¹, measurements were made in the 400–4000 cm⁻¹ spectral region. The FTIR analysis revealed bands of absorption that corresponded to protein carbonyl groups, suggesting that the AgNPs were probably capped and stabilized by the proteins in the neem extract. An X'Pert Pro diffractometer with Cu Kα radiation was used for X-ray diffraction (XRD) investigation in order to ascertain the nanoparticles' amorphous structure and size distribution. A scanning speed of 10°/min was used. An amorphous structure was confirmed by the XRD signals (Ugwuoke, 2023).

### Trial birds and research methodology

A total of 336, 1-day-old *C. coturnix japonica* chicks were purchased from the Avian Research and Training Center (ART center) at the University of Veterinary & Animal Sciences (UVAS), Lahore. These chicks were carefully transported and relocated to the Animal House at Government College University Faisalabad. The trial was conducted at Government College University Research Lab, Department of Zoology. The time period of trial was 45 days.

Birds were acclimatized for 10 days under controlled conditions. The temperature was 20-25°C, light period was 16hr day light/ 8hr dark and humidity was 70%. All the birds were kept under same temperature, humidity and hygienic conditions with proper care and handling. Birds had free access to commercial basal diet (Table 1) and fresh water all the time in wire cages. After ten days of acclimatization birds were randomly divided into 7 groups. Each group consists of 48 birds with 6 replicates (8 birds in each). The groups were as follows: 1st group was control group received only basal diet, 2nd group was administered with tartrazine low dose (Tz LD) (10 mg/kg) body weight, 3rd group received tartrazine high dose (Tz HD) (20mg/kg) body weight, 4th group received tartrazine low (10 mg/kg) and AgNPs low dose (4mg/kg) body weight, 5th group was administered with tartrazine low (10 mg/kg) and AgNPs high dose (8 mg/kg) body weight, 6th group received tartrazine high (20mg/kg) and AgNPs low dose (4mg/kg)body weight and 7th group administered with tartrazine high (20mg/kg)and AgNPs high dose (8mg/kg) body weight. All the birds administered with different doses of tartrazine and AgNPs via oral gavage. These dose levels were selected based on previous toxicological investigations and in reference to acceptable daily intake (ADI) considerations to simulate sub-chronic exposure scenarios exceeding normal dietary intake but useful for evaluating potential biological risks under intensive poultry production systems (Abbas et al., 2026). Tartrazine was dissolved in sterile distilled water, while the green-synthesized Ag-NPs were suspended in the same vehicle to ensure consistency across treatments. Oral gavage was employed to ensure accurate and uniform delivery of the intended doses and to minimize variation associated with feed intake differences among birds. Although this method differs from dietary inclusion, it enables controlled toxicological evaluation and clearer interpretation of dose-dependent genotoxic and histopathological responses, thereby providing mechanistic insights relevant to poultry health and food safety (Table 2).

**Table 1:** Major components and nutritional contents of Japanese quail diets.

Ingredients	Contents (%)
Yellow Corn	49.25
Soya bean meal	32.18
Starch	10.15
Limestone	6.50
Di-Calcium Phosphate	1.16
Salt (NaCl)	0.30
Alfalfa leaf powder	0.16
Vitamin and mineral premixture*	0.30
Calculated analysis*	
ME, Kcal/kg	2830
Crude protein	22.63
Crude Fiber	2.21
Ether Extract	2.19
Calcium	2.82
Phosphorous	0.33
Methionine + cystine	0.72
Methionine	0.44
Lysine	1.01

⁎ Each kg of vitamin and mineral mixture included the following: 10,000 IU of retinol, 3,500 IU of cholecalciferol, 35 IU of tocopherol, 1.67 mg of phylloquinone, 1.67 mg of thiamine, 2 mg of riboflavin, 3.67 mg of pyridoxine, 0.012 mg of cyanocobalamin, 6.67 mg of pantothenic acid, 16.7 mg of nicotinic acid, 1.67 mg of folic acid, 0.07 mg of biotin, 400 mg of choline chloride,0.03 mg Selenium, 133.4 g of Mg, 90 mg of Mn, 80 mg of Zn, 25 mg of Fe, 1.67 mg of Cu, and 0.8 mg of I.

**Table 2:** Effects of tartrazine and silver nanoparticles on various parameters 0f DNA in Quails.

Parameters	Control	Tz LD	Tz HD	Tz LD AgNPs LD	Tz LD AgNPs HD	Tz HD AgNPs LD	Tz HD AgNPs HD	P value
LHead (Mean ±SD)	26.33 ± 1.15^a^	5.00 ± 2.00^de^	3.67 ± 1.15^e^	10.33 ± 1.15^c^	17.00 ± 0.11^b^	7.67 ± 1.15^cd^	9.00 ± 2.00^c^	0.001⁎⁎⁎
LTail (Mean ±SD)	3.33 ± 0.58^b^	7.00 ± 2.00^ab^	9.00 ± 1.00^a^	4.67 ± 2.89^ab^	3.00 ± 0.01^b^	6.33 ± 0.58^ab^	5.67 ± 2.08^ab^	0.006⁎⁎
LComet (Mean ±SD)	29.66 ± 0.58^a^	12.00 ± 0.00^c^	12.67± 0.57^c^	15.00 ± 1.73^c^	20.00 ± 1.00^b^	14.00 ± 1.00^c^	14.67 ± 2.51^c^	0.001⁎⁎⁎
Head DNA (Mean ±SD)	99.04 ± 0.99^a^	25.33 ± 1.59^e^	11.39 ± 3.05^f^	67.42 ± 2.09^c^	93.44 ± 0.21^b^	48.90 ± 0.88^d^	66.21 ± 2.09^c^	0.001⁎⁎⁎
Tail DNA (Mean ±SD)	0.95 ± 0.99^f^	74.67 ± 1.59^b^	88.60 ± 3.05^a^	32.57 ± 2.09^d^	6.56 ± 0.11^e^	51.09 ± 0.88^c^	33.78 ± 2.09^d^	0.001⁎⁎⁎
TM (Mean ±SD)	0.03± 0.03^c^	4.49 ± 1.99^ab^	7.12 ± 1.74^a^	1.56 ± 1.07^bc^	0.19± 0.01^c^	2.05 ± 0.93^bc^	2.18 ± 1.07^bc^	0.001⁎⁎⁎
OTM (Mean ±SD)	0.13 ± 0.14^b^	2.84 ± 2.22^b^	5.90 ± 0.72^a^	1.67 ± 0.34^b^	0.54 ± 0.01^b^	1.60 ± 0.96^b^	1.87 ± 0.34^b^	0.001⁎⁎⁎

Tz=Tartrazine, AgNPs=Silver Nanoparticles, LD=Low Dose, HD=High Dose.

⁎⁎⁎ =highly significant.

⁎⁎ =significant.

### Characterization of AgNPs

AgNPs used in this study were synthesized and physicochemically characterized according to the standardized procedures described by Al-Khalaifah et al. (2025a). Characterization included X-ray diffraction (XRD), hydrodynamic diameter determined by dynamic light scattering (DLS), polydispersity index (PDI), and zeta potential to confirm the structural properties, size distribution, and stability of the nanoparticles. Detailed characterization data were previously reported by Al-Khalaifah et al. (2025a) and are referenced here to avoid redundancy.

### Blood sample collection

At the end of trial (day 45) 2-3 ml blood was drawn from the brachial vein of six birds, one from each replicate. For comet assay analysis all the samples were stored in lavender-topped tubes, by following the method described by Khalid et al. (2025). For chemical analysis blood samples were stored at −20°C.

### Tissue sample collection

Six birds, one from each replicate were slaughtered humanely and their organs removed carefully and then put in separate containers. All the samples were stored in formalin for further analysis.

### DNA damage examination

Single-Cell Gel Electrophoresis (SCGE), commonly referred to as the comet assay, was used to evaluate DNA damage induced by tartrazine and silver nanoparticle exposure. The procedure was performed following the protocol described by Al-Khalaifah et al. (2025a) with minor modifications. Briefly, cells embedded in agarose were lysed in a cold lysis buffer (pH 10) to remove cellular proteins, followed by DNA unwinding under alkaline conditions (pH > 13) for 20 min. Electrophoresis was conducted in alkaline buffer at **25** **V (≈0.8** **V/cm) and 300** **mA for 20** **min** at 4°C. After electrophoresis, slides were neutralized, stained, and examined under a fluorescence microscope. Image analysis was performed using Casp_1.2.3b1 software to quantify seven comet parameters: head length, tail length, total comet length, percentage of DNA in the head and tail, tail moment, and olive tail moment.

### Histopathological analysis

For histopathological evaluation, portions of the kidney, liver, and heart were preserved in 10% neutral buffered formalin and processed using standard procedures. Fixed tissues were embedded in paraffin, sectioned at 5-μm thickness using a rotary microtome, and stained with hematoxylin and eosin (H&E). For each organ, at least three distinct regions per sample were analyzed under a light microscope. Histopathological assessment was performed in a blinded manner, with the evaluator unaware of the treatment groups to minimize bias. Lesions were graded using a semi-quantitative scoring system for severity (0 = absent, 1 = mild, 2 = moderate, 3 = severe). Additionally, quantitative histomorphometric analyses were performed for relevant tissue features, including cell density, villus height, and tissue thickness, using ImageJ software. Representative microphotographs with scale bars were captured to document morphological changes and illustrate typical lesions for each treatment group.

### Statistical analysis

The effects of tartrazine and AgNP supplementation on genotoxicity and histopathological parameters in Japanese quails were analyzed using one-way analysis of variance (ANOVA) in SPSS software (version 21**).** The replicate cage was considered the experimental unit, as treatments were applied at the cage level and birds within each cage shared the same environmental and management conditions. Prior to ANOVA, data were checked for normality using the Shapiro–Wilk test and for homogeneity of variances using Levene’s test. When necessary, data were log-transformed to meet ANOVA assumptions. Cage mean values were used for statistical analysis. Significant differences among treatment means were determined using Tukey’s post hoc test, and actual p-values, effect sizes (η²), and 95% confidence intervals are reported to provide a comprehensive assessment of treatment effects. Statistical significance was declared at *p* < 0.05.

## Results

### Histopathological analysis of liver

**Fig. 1:**
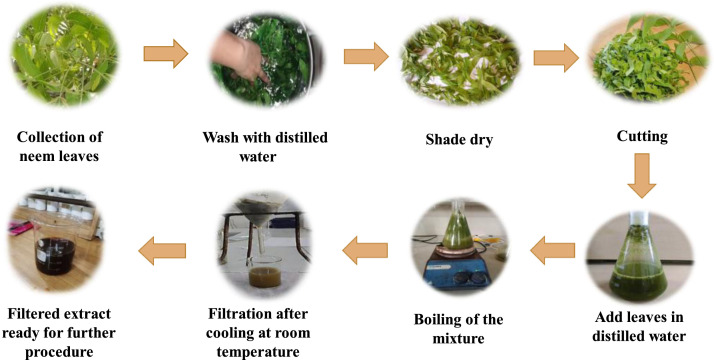
Preparation of plant extract for further procedure.

**Fig. 2:**
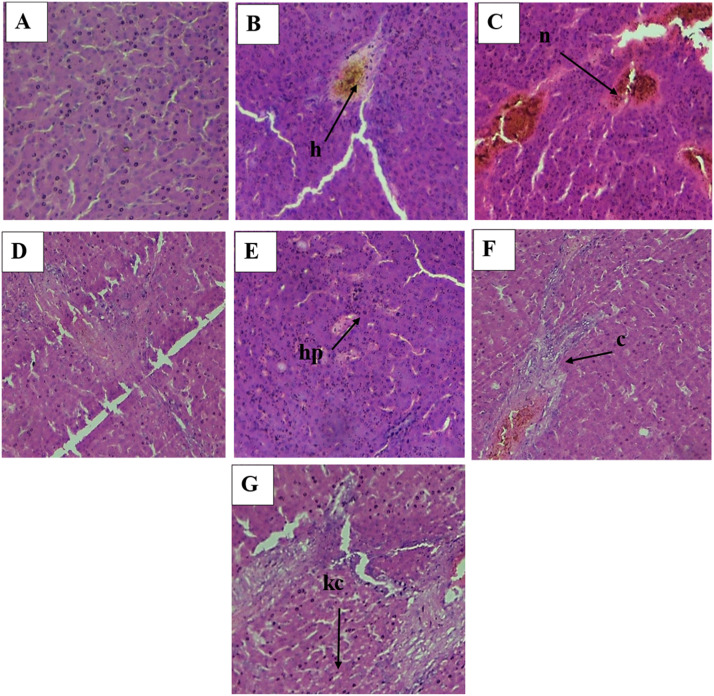
Histological (40X) sections of liver (A) Control Group (B) Tartrazine Low Dose Group (C) Tartrazine High Dose Group (D) Tartrazine Low and AgNPs Low Dose Group (E) Tartrazine Low and AgNPs High Dose Group (F) Tartrazine High and AgNPs Low Dose Group (G)Tartrazine High and AgNPs High Dose Group, h=hemorrhage, n=necrosis, sd=sinusoidal dilation, hp=hepatocyte, c=congestion, kc=Kupffer cells. Scale bar: 50 µm.

**Fig. 3:**
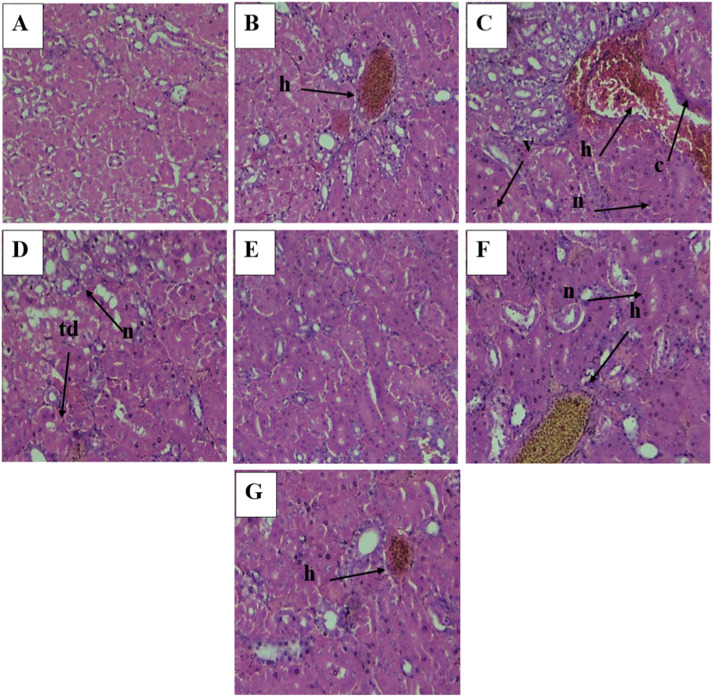
Histological (40X) sections of kidney (A) Control Group (B) Tartrazine Low Dose Group (C) Tartrazine High Dose Group (D) Tartrazine Low and AgNPs Low Dose Group (E) Tartrazine Low and AgNPs High Dose Group (F) Tartrazine High and AgNPs Low Dose Group (G)Tartrazine High and AgNPs High Dose Group, h=hemorrhage, n=necrosis, c=congestion, v=vacuolation, td=tubular dilation; Scale bar: 50 µm.

**Fig. 4:**
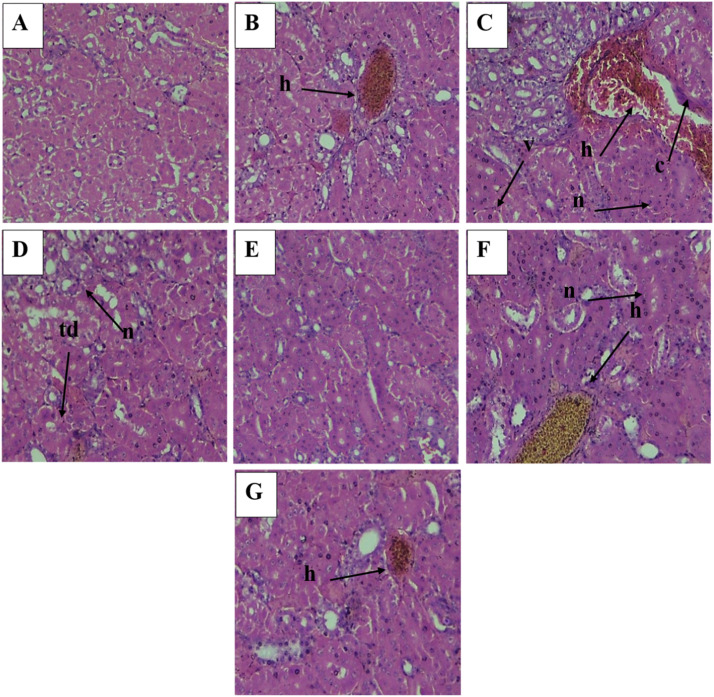
Histological (40X) sections of heart (A) Control Group (B) Tartrazine Low Dose Group (C) Tartrazine High Dose Group (D) Tartrazine Low and AgNPs Low Dose Group (E) Tartrazine Low and AgNPs High Dose Group (F) Tartrazine High and AgNPs Low Dose Group (G)Tartrazine High and AgNPs High Dose Group, e=edema, n=necrosis, i=inflammation, md=myofibrillar degeneration, v=vacuolation, m=myocyte, nf=necrotic fibers, Scale bar: 50 µm.

**Fig. 5:**
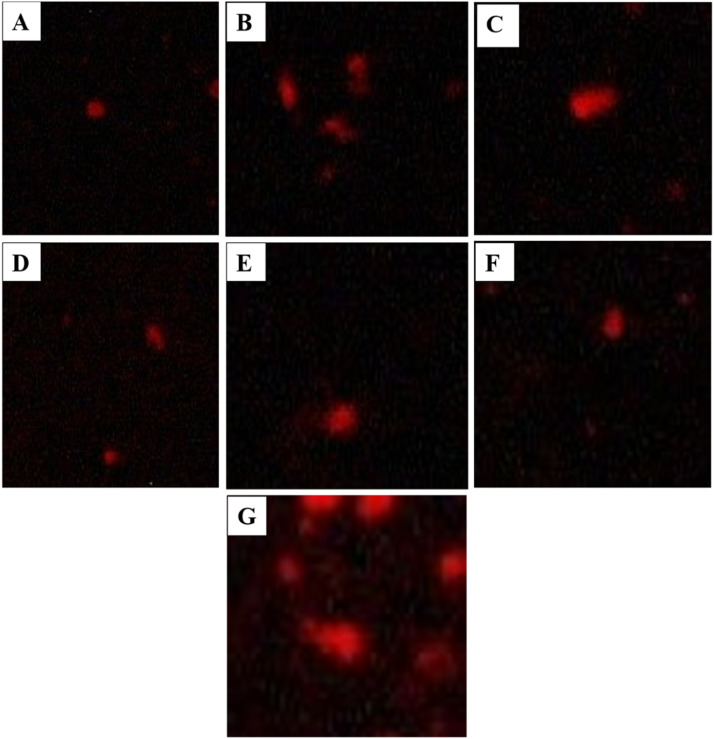
Microscopic images of Japanese quails blood samples after comet assay (A) Control Group (B) Tartrazine Low Dose Group (C) Tartrazine High Dose Group (D) Tartrazine Low and AgNPs Low Dose Group (E) Tartrazine Low and AgNPs High Dose Group (F) Tartrazine High and AgNPs Low Dose Group (G)Tartrazine High and AgNPs High Dose Group.

Fig. displayed the histological changes in the livers of Japanese quails from the control and different groups treated with tartrazine and green synthesized AgNPs. The control group (normal hepatocytes, sinusoidal spaces, no necrosis) maintained the liver's normal morphological and histological structure, constituted of large sized hepatocytes also known as polygonal cells. The tartrazine high dose group showed severe liver injury, hepatic sinusoidal congestion, and necrosis, dilated and irregular blood Sinusoids with hazy cell borders. The tartrazine low and AgNPs high dose (Tz LD AgNPs HD) group had normal cells, normal blood vessels, a little bit necrosis, and normal cell borders. Overall order of damage is as followed: Tz HD>Tz LD > Tz HD AgNPs LD > Tz HD AgNPs HD > Tz LD AgNPs LD > Tz LD AgNPs HD > Control (Fig. 1, Fig. 2, Fig. 3, Fig. 4, Fig. 5).

### Histopathological analysis of kidney

The kidney's normal tissue, which includes renal tubules and glomeruli and is encircled by simple cuboidal epithelial tissue, is seen in the control group. Compared to other groups, the tissues of the tartrazine-low and AgNPs-high group were almost normal and had significantly less damage. The high tartrazine group showed more severe pathological effects, such as increased bleeding, red blood cell stealth between the glomerulus and urinary tubules, and necrosis with pyknosis nuclei of renal epithelial cells. Bowman's space contracted and the basement membrane was damaged. Lumen stenosis eventually resulted from the severe swelling and degradation of the great majority of the epithelial cells lining the renal tubules.

### Histopathological analysis of heart

The control group's myocardium displayed the normal histological architecture, including anastomosing muscle fibers with centrally placed oval vesicular nuclei, acidophilic sarcoplasm, and longitudinally striated branching. There are little intercellular gaps between them. Myocardial fibers in the tartrazine low and AgNPs high dose groups have a regular arrangement and a usual nucleus. There were clear borders and a regular arrangement of cardiac muscle fibers. Tartrazine high groups exhibited Brown atrophy, necrosis, and severe fiber rupture cardiac fiber disarray and fiber lysis. Congestion was also seen in both high categories. Muscle fibers in the high tartrazine group were described as being loose, asymmetrical, shorter, and even ruptured.

### Comet assay analysis

ANOVA was used to examine all comet parameters across groups that received varying dosages of tartrazine and Ag-NPs. The mean values of LHead, LTail, LComet, HeadDNA, TailDNA, TM, and OTM showed significant differences (*P* < 0.05). A post-Hoc Tukey test revealed that highest values of LTail (9.00 ± 1.00) and tailDNA (88.60 ± 3.05) were observed in group treated with high dose of tartrazine (20mg/kg), showed the greatest damage. Highest values of LHead (17.00 ± 0.00) and headDNA (93.44 ± 0.00) were observed in group treated with tartrazine LD and AgNPs HD, show less damage. Overall birds treated with different doses showed different results according to the treatment. The order of damage from high to low is as follows: Tz HD>Tz LD > Tz HD AgNPs LD > Tz HD AgNPs HD > Tz LD AgNPs LD > Tz LD AgNPs HD > Control.

## Discussion

This study focusses on the use of silver nanoparticles made from *Azadiracta indica* extract as a new, sustainable, and protective agent against genotoxic effects and structural and functional abnormalities caused by tartrazine in Japanese quails (Soleimani et al., 2019). The increasing usage of green synthesis from more than 65 medicinal plants is highlighted by recent studies, underscoring the importance of biocompatible and environmentally friendly nanomaterials for upcoming biomedical applications (Saeed et al., 2023). Additionally, the characterization results verified that Ag nanoparticles were successfully synthesized. Presence of silver was confirmed using X-ray diffraction analysis, FTIR, and UV visible spectroscopy (Al-Khalaifah et al., 2025a). However, it is important to recognize that silver nanoparticles possess a dual biological nature, as previous studies have reported both protective and potentially toxic effects depending on dose, exposure duration, and biological system. Therefore, interpretation of their protective role should be made cautiously.

According to the current study, tartrazine intake seriously harms the tissues of the liver, kidney, and heart. An indicator of potential tissue injury is elevated blood levels of liver enzyme activity. These findings are consistent with those of previous researchers who linked hepatocellular damage to comparable alterations in liver function (Althumairy, 2025). The liver is a sizable, intricate organ that is well suited for its crucial function in the metabolism of fat, protein, and carbohydrates. The liver's entire enzyme system aids in the synthesis and metabolism of chemicals. When it is disturbed, there may be more release into the blood, which could result in tissue damage and other severe problems (Giannini et al., 2005). In this study, kidney tissues are more damaged in group administered with high dose of tartrazine as compared to other groups. Tartrazine administration increases the creatinine and urea level that cause damage to kidney and many more problems Azu et al., 2010). Since tartrazine metabolism produces free radicals and raises oxidative stress, the histological alterations seen in this study may be the consequence of oxidative damage to the heart tissues caused by tartrazine (Iroh et al., 2020). Research of Uyota et al. (2020), which showed a non-significant drop in SOD and a large increase in MDA, supports this. Superoxide Dismutase (SOD) is an essential antioxidant enzyme in the heart that neutralizes dangerous superoxide radicals, shielding heart tissue from oxidative stress, inflammation, fibrosis, and damage from events like heart attacks. Malondialdehyde (MDA) is a key indicator of oxidative stress, a byproduct of lipid peroxidation (damage to cell membranes by free radicals), indicating cellular damage from harmful reactive oxygen species (ROS). Significant oxidative damage is indicated by elevated MDA levels in the blood, which are associated with worse outcomes in atherosclerosis, heart failure, and many other disorders (Zheng et al., 2008). It should be noted that the study did not measure oxidative stress biomarkers. Therefore, previous mechanistic interpretations regarding ROS, SOD, or MDA are speculative. Statements regarding antioxidant activity and ROS scavenging have been toned down to reflect this limitation.

In this study, heart tissues highly damaged in the group administered with high dose of tartrazine as compared to the groups treated with tartrazine along with the green silver nanoparticles because AgNPs mitigate the effects caused by tartrazine. However, because AgNP-only control groups were not included, it cannot be definitively concluded whether the observed improvements were solely due to protective effects of AgNPs or whether AgNP exposure itself contributed to biological responses. This represents an important limitation of the experimental design and has now been acknowledged accordingly. The strong antioxidant activity of AgNPs is one of the study's main conclusions. These alterations show that AgNPs successfully reduce oxidative stress and maintain cellular redox equilibrium. The phytochemical components involved in the creation of nanoparticles, which promote endogenous defense mechanisms is probably responsible for this antioxidant response (Zhang et al., 2019). Silver nanoparticles made from various botanical sources, such as garlic and onion peel extracts, have been shown to exhibit comparable cytoprotective and antioxidant properties (Ali et al., 2019). In this study, group administered with low dose of tartrazine and high dose of AgNPs has less damage in liver, kidney and heart tissues due to antioxidant and other protective properties of AgNPs. Although causality cannot be fully separated from nanoparticle-specific effects due to the absence of AgNP-only treatments. Overall damage order of histopathology of liver, kidney and heart is as followed: Tz HD > Tz LD > Tz HD AgNPs LD > Tz HD AgNPs HD > Tz LD AgNPs LD > Tz LD AgNPs HD > Control.

The comet assay is regarded as a reliable biomarker of the genotoxicity of food additives and is well-known for its sensitive, quick, and affordable detection of DNA breaks. Because it is a carrier of harmful contaminants, whole blood was selected. Since tartrazine belongs to the class of azo dye food colorants, intestinal flora breaks it down into aromatic amines, which can produce reactive oxygen species (ROS), which are known to harm DNA (Bansal et al., 2005). DNA damage was considerably more severe in samples exposed to tartrazine than in those treated with tartrazine coupled with Ag-NPs or the control group, according to recent pathological studies investigating the genotoxic effects of tartrazine and Ag-NPs, which were validated by comet assays. Regarding the genotoxicity of artificial coloring, the results showed that tartrazine damaged blood DNA as measured by the comet assay. The direct interaction of tartrazine with nuclear DNA is most likely the cause of this genotoxic impact (Himri et al., 2012). Green-synthesized silver nanoparticles were associated with reduced DNA damage in treated groups; however, these findings should be interpreted cautiously and considered indicative of a potential modulatory effect rather than definitive antioxidant protection.

## Conclusion

This work offers important new information about tartrazine's genotoxic and histopathological effects in Japanese quail (*Coturnix coturnix japonica*). The study demonstrated that tartrazine cause severe damage to different tissues of body and cause DNA damage as compared to other groups that were administered with tartrazine along with AgNPs because as an antioxidant, green produced silver nanoparticles are effective against tartrazine by mitigating the effects induced by tartrazine. The long-term effects of tartrazine and Ag-NPs supplementation on quail health should be investigated in future studies. It will be crucial to look at cost-effectiveness, optimal dosage, and interactions with other dietary components.

## Funding

Not applicable

## Ethical statement

This study was approved by the ethical committee of, the department of Zoology, Government college University, Faisalabad Paksitan (No 460/DoZ/2024).

## Data availability statement

Data is available from the corresponding author upon reasonable request.

## AI

AI (chatgpt) has been used for English language.

## CRediT authorship contribution statement

**Samra:** Investigation. **Shabana Naz:** Supervision, Conceptualization. **Maryam Fatima:** Methodology, Investigation. **Fiza Abbas:** Visualization, Validation. **Ulfat Zahra:** Investigation, Data curation. **Rasha Alonaizan:** Writing – review & editing. **Hafsa Saeed:** Formal analysis, Data curation. **Sania Satti:** Visualization, Validation, Formal analysis. **Rifat Ullah Khan:** Writing – review & editing, Writing – original draft, Visualization, Investigation. **Ala Abudabos:** Writing – review & editing, Writing – original draft. **Ali R. Al Sulaiman:** Writing – review & editing. **Raed Al-Atiyat:** Writing – review & editing. **Sohail Ahmad:** Resources, Funding acquisition. **Ibrahim A. Alhidary:** Resources, Funding acquisition.

## Disclosures

Authors declare no conflict of interest.
